# Efficacy of N-acetylcysteine plus pirfenidone in the treatment of idiopathic pulmonary fibrosis: a systematic review and meta-analysis

**DOI:** 10.1186/s12890-023-02778-w

**Published:** 2023-11-29

**Authors:** Xiu-Li Zhang, Ying Cao, Bo Zheng

**Affiliations:** 1 Medical Department, Chengdu Qingbaijiang District People’s Hospital, No 9, Fenghuang East Fourth Road, Qingbaijiang District, Chengdu, 610300 China; 2 Department of Infectious Diseases, Chengdu Xinjin District People’s Hospital, No 149, Wujin West Road, Xinjin District, Chengdu, 611430 China

**Keywords:** N-acetylcysteine, Pirfenidone, Idiopathic pulmonary fibrosis, Efficacy, Safety, Meta-analysis

## Abstract

**Background:**

Numerous studies have demonstrated the potential of pirfenidone to enhance the prognosis of patients afflicted with idiopathic pulmonary fibrosis (IPF). Although N-acetylcysteine (NAC) is utilized as an antioxidant in IPF treatment, the combination of NAC and pirfenidone has produced inconsistent outcomes in certain studies. To assess the clinical effectiveness and safety of NAC plus pirfenidone (designated as the treatment group) versus pirfenidone monotherapy (designated as the control group), we conducted a systematic review and meta-analysis of randomized controlled trials (RCTs).

**Methods:**

RCTs of NAC plus pirfenidone were reviewed searching from databases and networks of unpublished and published studies in any language. Using pair-wise meta-analysis, changes in pulmonary function test (PFT) parameters and safety were evaluated.

**Results:**

Two independent reviewers selected and obtained data from 5 RCTs (*n* = 398), comprising 1 study from Japan, 1 from Europe, and 3 from China. NAS plus pirfenidone as compared to pirfenidone monotherapy for IPF may not reduce the incidence of skin effects(RR 1.26 [95%CI 0.64 to 2.45]) and mortality(RR 0.35 [95%CI 0.07 to 1.68])(both moderate certainty). NAS plus pirfenidone as compared to pirfenidone monotherapy for IPF may not reduce the incidence of at least one side effects(RR 1.00 [95%CI 0.84 to 1.19]; low certainty),severe side effects(RR 0.67 [95%CI 0.30 to 1.47]; low certainty) and gastrointestinal effects(RR 0.67 [95%CI 0.41 to 1.09]; low certainty) with possibly no effect in Δ%DLco(SMD -0.17 [95%CI -0.15 to 0.48]; low certainty). Meanwhile, the effect of NAS plus pirfenidone as compared to pirfenidone monotherapy on ΔFVC(SMD 0.18 [95%CI -0.68 to 1.05]), Δ%FVC(SMD -2.62 [95%CI -5.82 to 0.59]) and Δ6MWT(SMD -0.35 [95%CI -0.98 to 0.28]) is uncertain(extremely low certainty).

**Conclusion:**

Moderate certainty evidence suggests that NAS plus pirfenidone, compared to pirfenidone monotherapy for IPF, does not reduce the incidence of skin effects and mortality.

**Supplementary Information:**

The online version contains supplementary material available at 10.1186/s12890-023-02778-w.

## Background

IPF is commonly associated with fibrosing interstitial pneumonia and a poor prognosis [1–3]. Among adults over 65 years of age, Schafer SC et al. [4] reported 494 cases per 100,000 people, which is double the rate observed a decade ago. Patients diagnosed with IPF typically experience a median survival time of 2–5 years, and quite a few of them are not in the early stage when they see a doctor, resulting in a marked deterioration in their quality of life [5, 6]. However, drug treatment for IPF is not effective enough, and there is a pressing need for additional treatment modalities [1–4]. Pirfenidone has been shown to regulate transforming growth factors and fibroblasts, thereby producing antioxidant and antifibrotic effects, as well as regulating reactive oxygen species metabolism [7, 8]. Multiple RCTs have provided evidence that pirfenidone can effectively decelerate the progression of lung function and extend progression-free survival [9, 10]. NAC, an antioxidant and scavenger of oxygen free radicals, serves as a precursor of glutathione and can collaborates with catalase to decompose hydrogen peroxide into oxygen and water [11]. The addition of NAC to azathioprine and prednisone has been demonstrated to postpone the decline of lung function in the IFIGENIA study [11, 12].

Several case–control and cohort studies conducted in Japan, Germany, and China have demonstrated the efficacy of NAC plus pirfenidone for the treatment of IPF [13–17]. However, a number of high-quality RCTs, such as those conducted by Sakamoto S et al. and PANORAMA research, have revealed that the combination therapy does not offer superior efficacy compared to pirfenidone monotherapy for IPF treatment. Furthermore, the combination therapy has been found to increase the incidence of adverse effects [18, 19].

To mitigate the impact of confounding variables, and building upon the robust findings of Sakamoto S et al. [18], this study exclusively undertook a meta-analysis of RCTs pertaining to the use of NAC plus pirfenidone versus pirfenidone monotherapy for the management of IPF.

## Methods

### Data resources and literature search

The present systematic review and meta-analysis has been duly registered in PROSPERO (http://www.crd.york.ac.uk/PROSPERO), with the registration number CRD42023417130. Furthermore, the Preferred Reporting Items for Systematic Reviews and Meta-Analyses (PRISMA) statement and PRISMA 2020-checklist were employed to carry out this systematic review and meta-analysis (Tables S1 and S2) [20]. A highly experienced librarian was enlisted to carry out the search strategy, which spanned from the inception of the database to April 5, 2023, and encompassed EMBASE, Cochrane Library, PubMed, Web of Science, and various Chinese databases (namely, Wanfang Database, CNKI, and VIP database). Additionally, searches were conducted on The EU Clinical Trials Register and clinicaltrials.gov to identify potential studies. The search terms employed were “N-acetylcysteine”, “acetylcysteine”, “pirfenidone”, “pulmonary fibrosis”, “idiopathic pulmonary fibrosis”, and “IPF” for all studies. For example, our search terms in Pubmed are:((N-acetylcysteine[All Fields]) OR (acetylcysteine[All Fields])) AND (pirfenidone[All Fields]) AND ((pulmonary fibrosis[All Fields]) OR (idiopathic pulmonary fibrosis[All Fields]) OR (IPF[All Fields])).We strictly follow the above search keywords to search, and there are no other restrictions in the database search.

### Research selection

Inclusion criteria: The present study incorporated RCTs that evaluated the efficacy of pirfenidone plus NAC or pirfenidone monotherapy for the management of IPF. Exclusion criteria: Case–control studies, cohort studies, case reports, and other types of studies must be excluded. Additionally, we conducted comprehensive searches across pertinent networks but studies that do not meet the criteria must be excluded. Two independent researchers conducted title and abstract screening, and articles that met the eligibility criteria were subjected to full-text screening and ultimately included in the analysis.

### Data extraction and quality assessment

Two researchers (XLZ and BZ) extracted the data independently. In the event of discrepancies, a third researcher is tasked with identifying and resolving them. When necessary, we consult pertinent authors and obtain relevant data. Any studies deemed unsuitable or incomplete are excluded from analysis. The parameters were extracted from qualifying studies: (1) Patient demographics, time of diagnosis, drug utilization, dosage, and duration; (2) Primary outcomes pertaining to changes in lung function (ΔFVC, ΔFVC%, Δ6MWT, ΔDLco%); and (3) Secondary outcomes: Incidence rates of at least one side effects, severe side effects, gastrointestinal effects, skin side effects, and mortality.

### Risks of bias

Two researchers conducted independent assessments of the risk of bias in RCTs using the Cochrane Collaboration risk of bias tool [21]. The risk of bias was assessed across various domains, including random sequence generation, allocation concealment, blinding of participants and personnel, incomplete outcome data, selective reporting, and other biases. The ratings were categorized as either (I) low risk of bias, (II) probably low risk of bias, (III) probably high risk of bias, or (IV) high risk of bias. In the event of any discrepancies, a third researcher was consulted to facilitate resolution. The Cochrane Collaboration risk of bias tool was employed to assess five RCTs, revealing that 4 of them were deemed high risk, with the exception of the article authored by Behr J et al. (Table 3) [18, 19, 22–24].

### Evidence quality GRADE evaluation

Two researchers independently and repeatedly assessed the certainty of the evidence using the GRADE approach (Table 1) [25, 26]. The evidence quality was assessed across various domains, including risk of bias, inconsistency, indirectness, imprecision, and publication bias. The ratings were categorized as either (I)extremely low certainty, (II) low certainty, (III) moderate certainty, or (IV) high certainty [27].

**Table 1:** GRADE quality assessment criteria

Study design	Initial quality of a body of evidence	Quality of evidence	Lower if	Higher if
RCT	High	High	Risk of Bias	Large effect
		Moderate	-1 Serious	+ 1 Large
		low	-2 Very serious	+ 2 Very large
		Extremely low	Inconsistency	Dose response
			-1 Serious	+ Evidence of a gradient
			-2 Very serious	All plausible residual confounding
			Indirectness	+ 1 Would reduce a demonstrated effect
			-1 Serious	+ 1 Would suggest a spurious effect if no effect was observed
			-2 Very serious	
			Publication bias	
			-1 Likely	
			-2 Very likely	

### Statistical analysis

The statistical analysis was conducted utilizing RevMan software (Version 5.0.1) [28]. The primary outcomes of this analysis encompassed ΔFVC, Δ%FVC, Δ6MWT, Δ%DLco, incidence of at least one side effects, incidence of severe side effects, incidence of gastrointestinal effects, incidence of skin side effects, and mortality from all causes in the treatment of IPF with pirfenidone plus NAC versus pirfenidone monotherapy. Mean and standard deviation were employed to express all data, while frequency and percentage were used to summarize the classification outcomes. Binary results were expressed using relative risk (RR) and 95% confidence intervals. Results of PFT parameters and other continuous variables were presented using Standardized mean difference (SMD). The level of heterogeneity was tested using the I^2^ statistic, with values exceeding 25%, 50%, and 75% indicating low, moderate, and high heterogeneity, respectively. All models apply random effects due to the inherent differences in populations. Unless otherwise specified, *P* < 0.05 is considered statistically significant [29, 30].

## Results

### Literature search and risk score

**Fig. 1:**
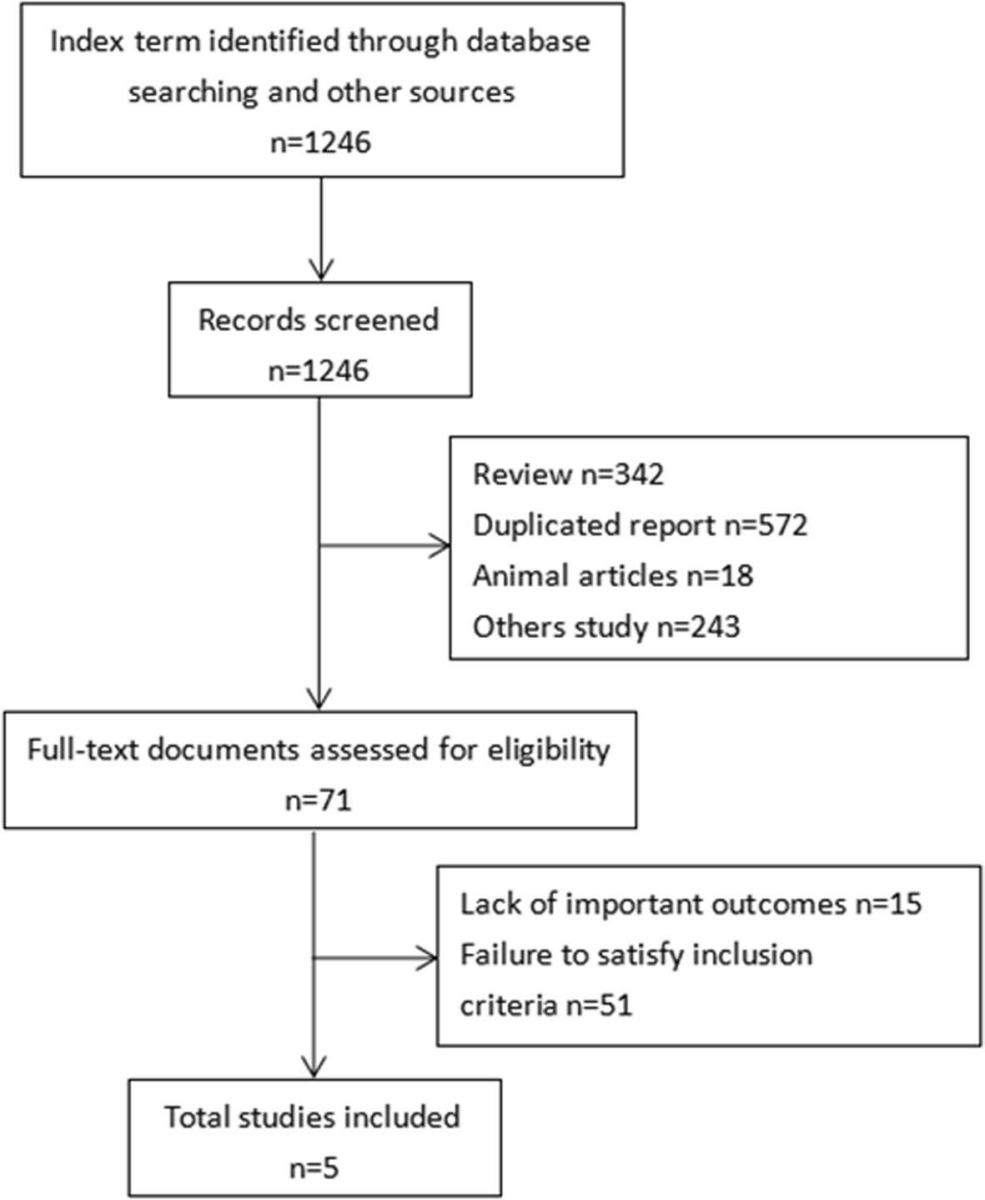
Flowchart of the article evaluation process in this meta-analysis

A total of 1246 relevant studies were searched and identified. Through the examination of titles and abstracts, 71 highly relevant articles were found. Following a meticulous full-text screening of the remaining 71 articles, 5 RCT articles were ultimately identified (Fig. 1) [18, 19, 22–24]. Notably, these 5 articles were conducted in various regions, including 1 in Japan, 1 in Europe, and 3 in China. 3 articles from China were published in Chinese, while the remaining articles were published in English. 2 were sourced from the EMBASE database [18, 19], and 3 were obtained from the CNKI database [22–24]. The analysis encompassed a total of 398 individuals, with 196 in the treatment group and 202 in the control group (Table 2).

**Table 2:** Basic information of 5 RCTs

Reference	Location	Study Design	Date of Trial	TG/CG (n)	Age (years, TG/CG)	TG(NAC + PFD,mg/d)	CG(PFD,mg/d)	Duration of Treatment	Outcomes
Sakamoto (2021) [18]	Japan	M,RCT	Jun 2015—Jun 2018	34/36	71.0 ± 7.3/73.3 ± 7.0	704.8 (inhaled) + 1200–1800	1200–1800	48 weeks	①⑤⑥⑦⑧⑨
Behr (2016) [19]	Europe (8 countries)	M,DB,RCT	Jun 2013—Feb 2015	60/62	66·7 ± 8.0/66·7 ± 6.2	1800 + 1602–2403	1602–2403 + placebo	24 weeks	①②③④⑤⑥⑦⑧⑨
Wen (2019) [22]	China	RCT	Nov 2012—Nov 2015	43/43	56.24 ± 10.2/55.63 ± 10.54	1800 + 1200	1200	6 month	①④⑥⑨
Zhou (2021) [23]	China	RCT	Jan 2018—Jan 2020	38/40	65.81 ± 8.76/66.45 ± 9.23	1800 + 600–1800	600–18	6 month	①⑤⑥⑦⑧⑨
Zhao (2023) [24]	China	RCT	Mar 2019—Mar 2022	21/21	64.3 ± 9.2/61.1 ± 8.8	600(inhaled) + 600–1800	600–1800	24 weeks	①③④⑤⑥⑦⑧⑨

*CCT* Case–control trial, *CS* Cohort study, *RCT* Randomized controlled trial, *NAC* N-acetylcysteine, *PFD* Pirfenidone, *M* Multicenter, *DB* Double-blind, *CG* Control group, *TG* Treatment group

① ΔFVC Changes in forced vital capacity

② ΔFVC% Changes in forced vital capacity percent predicted

③ Δ6MWT Changes in 6-min walking test distance

④ ΔDLco% Changes in percentage of predicted carbon monoxide diffusing capacity

⑤ At least one side effects

⑥ Severe side effects

⑦ Gastrointestinal effects

⑧ Skin side effects

⑨ Mortality rate

### Effect of NAC plus pirfenidone on outcomes of IPF

**Fig. 2:**
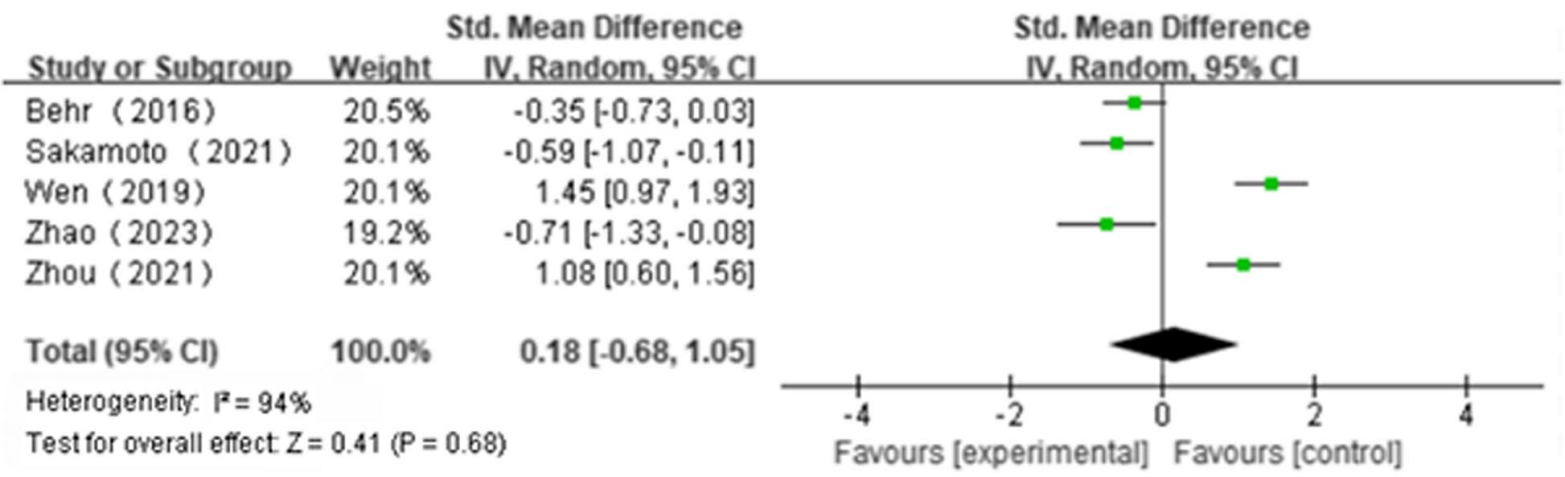
ΔFVC forest plot

**Fig. 3:**
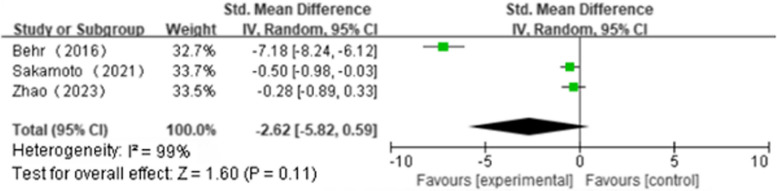
Δ%FVC forest plot

**Fig. 4:**
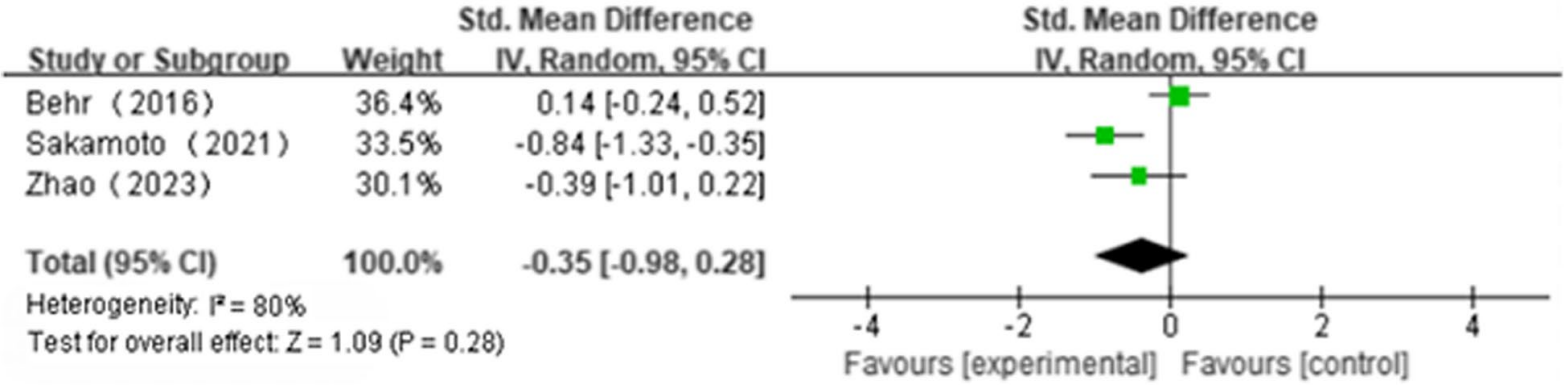
Δ6MWT forest plot

**Fig. 5:**
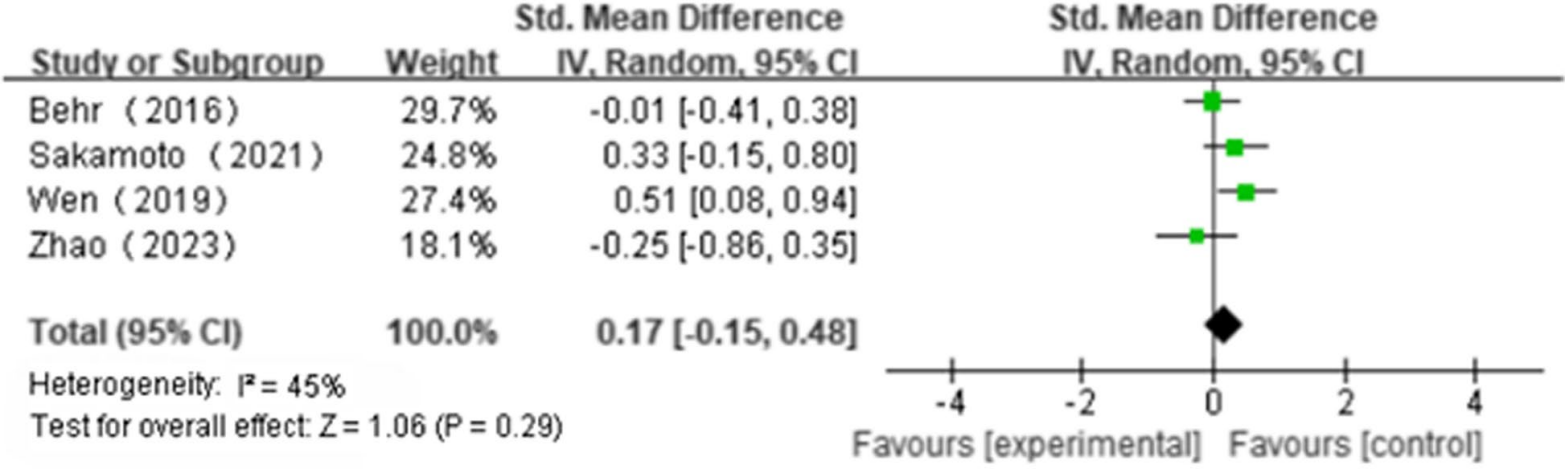
Δ%DLco forest plot

Five articles reported ΔFVC(NAC + PFD:*n* = 187, PFD alone:*n* = 195); We found that NAS plus pirfenidone may not improve ΔFVC as compared to pirfenidone monotherapy for IPF with high heterogeneity(SMD 0.18; 95%CI -0.68 to 1.05, *P* = 0.68, I^2^ = 94%; extremely low certainty) (Fig. 2 and Table 4) [18, 19, 22–24]. Three articles reported Δ%FVC(NAC + PFD:*n* = 106, PFD alone:*n* = 112); We found that NAS plus pirfenidone may not improve Δ%FVC as compared to pirfenidone monotherapy for IPF with high heterogeneity(SMD -2.62, 95%CI -5.82 to 0.59, *P* = 0.11, I^2^ = 99%; extremely low certainty) (Fig. 3 and Table 4) [18, 19, 24]. Three articles reported Δ6MWT(NAC + PFD:*n* = 107, PFD alone:*n* = 112); We found that NAS plus pirfenidone may not improve Δ6MWT as compared to pirfenidone monotherapy for IPF with high heterogeneity(SMD -0.35, 95% CI -0.98 to 0.28, *P* = 0.28, I^2^ = 80%; extremely low certainty) (Fig. 4 and Table 4) [18, 19, 24]. Four articles reported Δ%DLco(NAC + PFD:*n* = 143, PFD alone:*n* = 154); We found that NAS plus pirfenidone may not improve Δ%DLco as compared to pirfenidone monotherapy for IPF with moderate heterogeneity(SMD -0.17, 95% CI -0.15 to 0.48, *P* = 0.29, I^2^ = 45%; low certainty) (Fig. 5 and Table 4) [18, 19, 22, 24].

### Safety profile of NAC plus pirfenidone in the treatment of IPF

**Fig. 6:**
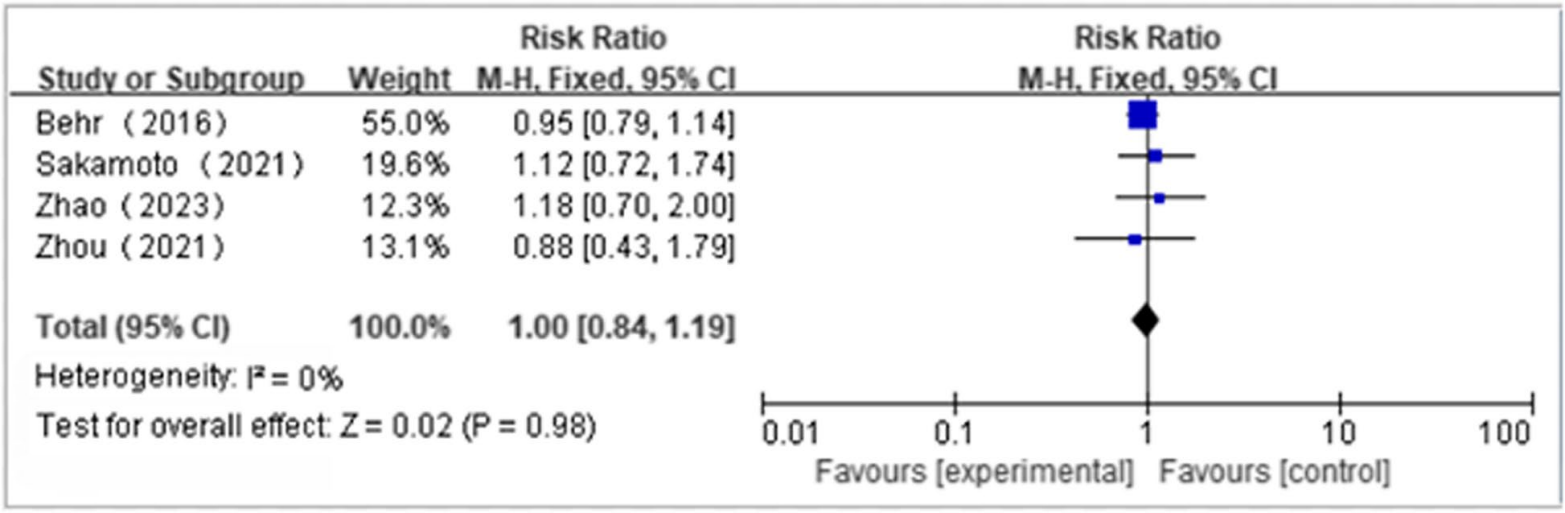
Side effects forest plot

**Fig. 7:**
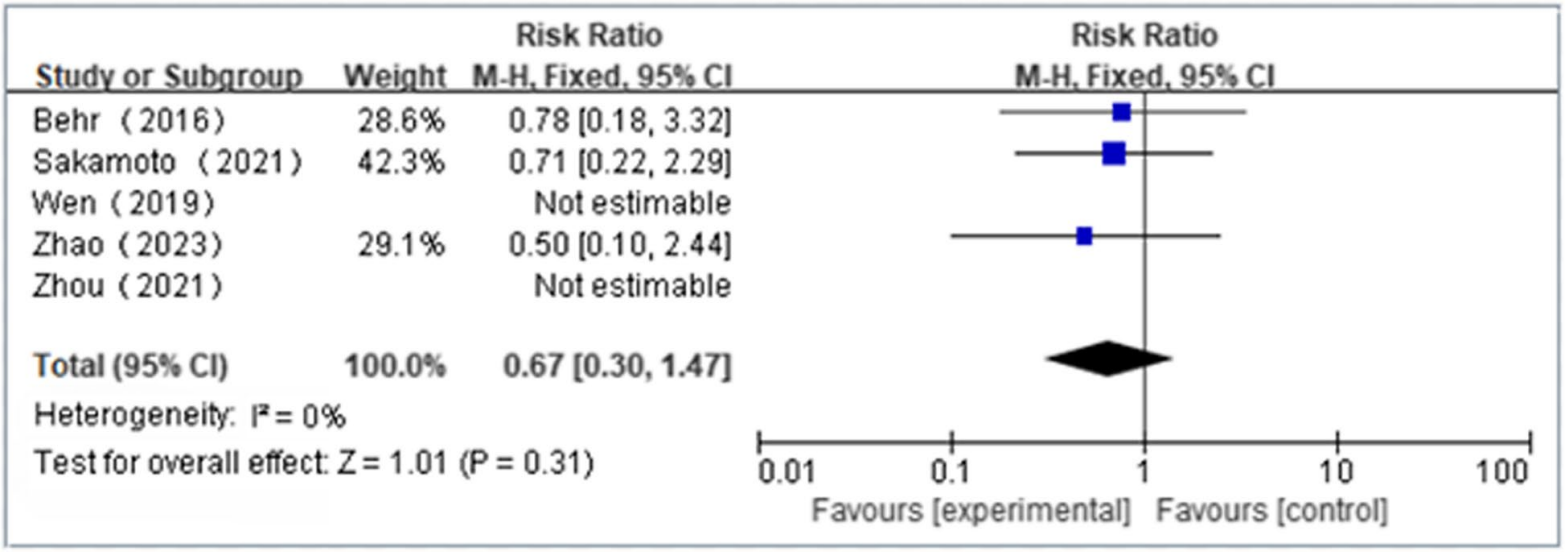
Severe side effects forest plot

**Fig. 8:**
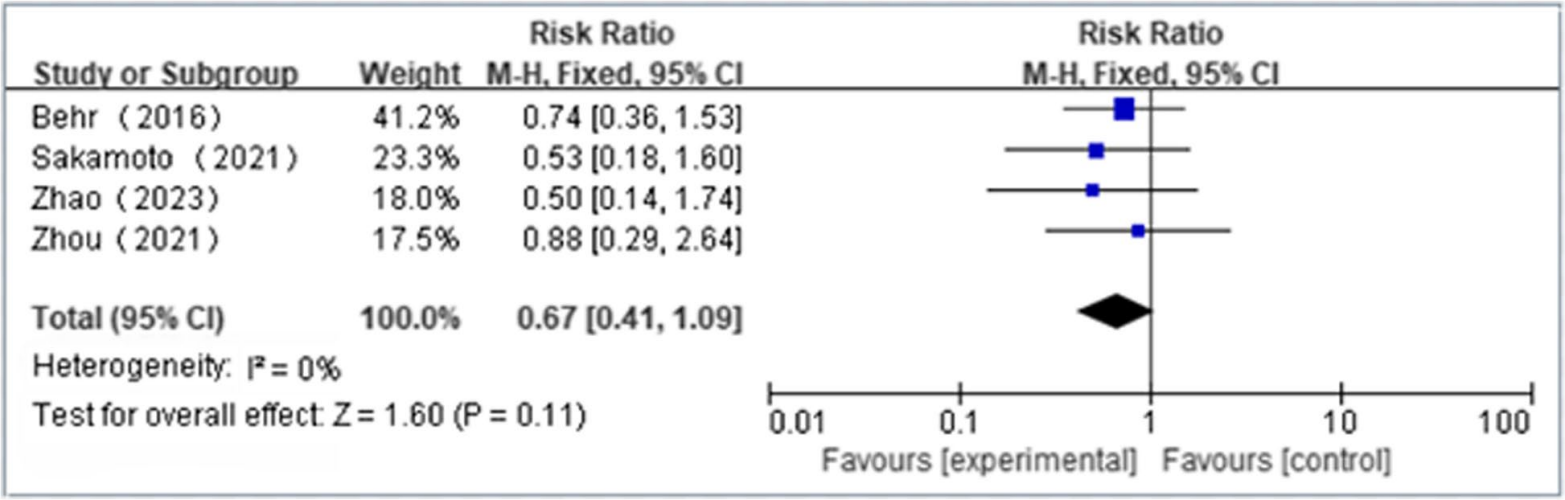
Gastrointestinal effects forest plot

**Fig. 9:**
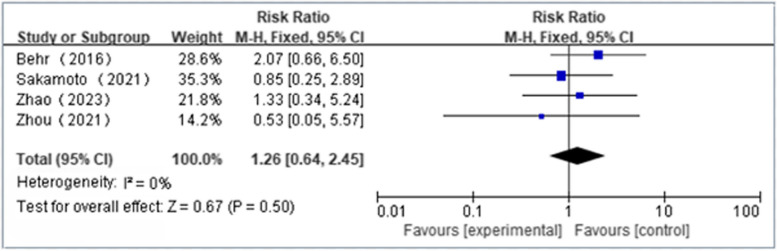
Skin effects forest plot

**Fig. 10:**
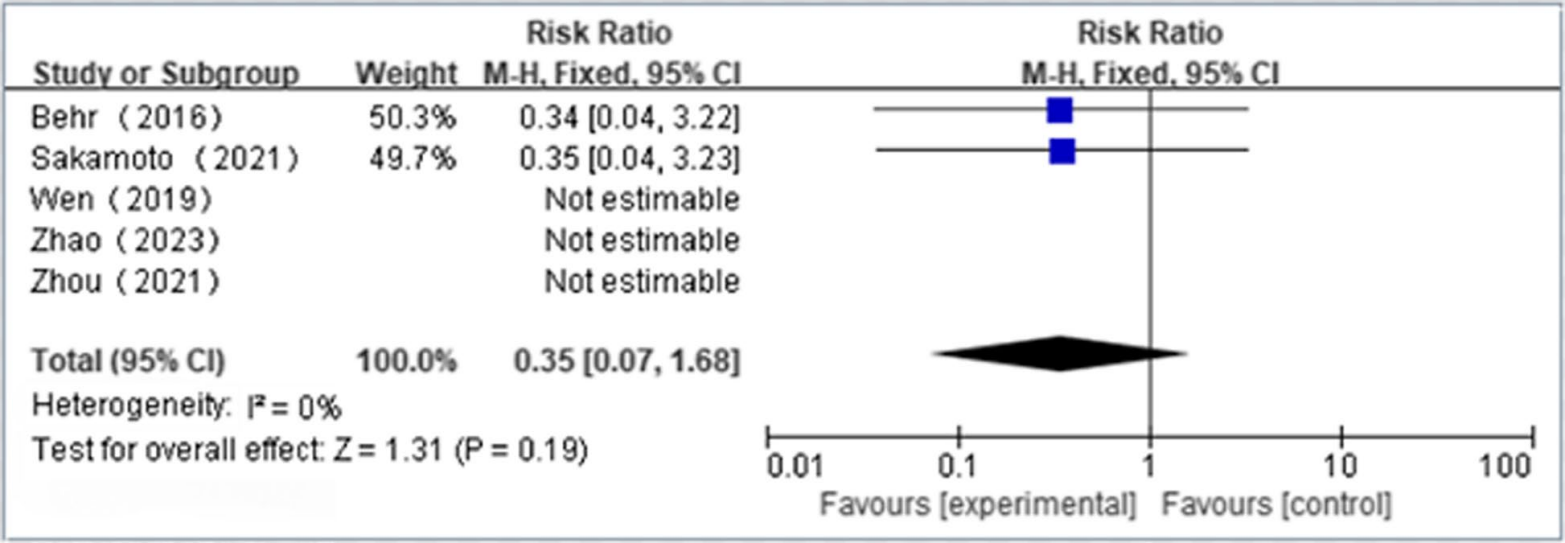
Mortality rates forest plot

Four articles reported the incidence of at least one side effect(NAC + PFD:*n* = 153, PFD alone:*n* = 159); We found that NAS plus pirfenidone may not reduce the incidence of at least one side effects as compared to pirfenidone monotherapy for IPF with low heterogeneity(RR 1.00, 95%CI 0.84 to 1.19, *P* = 0.98, I^2^ = 0%; low certainty) (Fig. 6 and Table 4) [18, 19, 23, 24]. Five articles reported the incidence of severe side effects(NAC + PFD:*n* = 196, PFD alone:*n* = 200); We found that NAS plus pirfenidone may not reduce the incidence of severe side effects as compared to pirfenidone monotherapy for IPF with low heterogeneity(RR 0.67, 95%CI 0.30 to 1.47, *P* = 0.31, I^2^ = 0%; low certainty) (Fig. 7 and Table 4) [18, 19, 22–24]. 4 articles reported the incidence of gastrointestinal effects(NAC + PFD:*n* = 153, PFD alone:*n* = 159); We found that NAS plus pirfenidone may not reduce the incidence of gastrointestinal effects as compared to pirfenidone monotherapy for IPF with low heterogeneity(RR 0.67, 95%CI 0.41 to 1.09, *P* = 0.11, I^2^ = 0%; low certainty) (Fig. 8 and Table 4) [18, 19, 23, 24]. 4 articles reported the incidence of skin effects(NAC + PFD:*n* = 153, PFD alone:*n* = 159); We found that NAS plus pirfenidone may not reduce the incidence of skin effects as compared to pirfenidone monotherapy for IPF with low heterogeneity(RR 1.26, 95%CI 0.64 to 2.45, *P* = 0.50, I^2^ = 0%; moderate certainty) (Fig. 9 and Table 4) [18, 19, 23, 24]. Five articles reported the incidence of mortality(NAC + PFD:*n* = 196, PFD alone:*n* = 202); We found that NAS plus pirfenidone may not reduce the incidence of mortality as compared to pirfenidone monotherapy for IPF with low heterogeneity(RR 0.35,95%CI 0.07 to 1.68, *P* = 0.19, I^2^ = 0%; moderate certainty) (Fig. 10 and Table 4) [18, 19, 22–24].

### Bias and sensitivity analysis

If the funnel plot of the meta-analysis describes that most studies are located in the upper region of the “inverted funnel” and show a rough symmetry, it indicates that publication bias is not significant, and vice versa, publication bias is significant. According to the findings from the funnel plot analysis, no substantial bias was observed in the publication of ΔFVC, Δ6MWT, Δ%DLco, skin effects, and mortality rates. However, a significant bias was detected in the publication of Δ%FVC, side effects, severe side effects, and gastrointestinal effects. However, the number of articles is limited, which limits the significance of funnel plot to some extent. There were only 5 relevant RTC trials, 2 for inhalation administration and 3 for oral administration. In order to reduce heterogeneity and increase sensitivity, oral administration studies was performed for analysis, and the forest plots can be found in the Supplement Material (Figs. S10, S11, S12, S13, S14, S15 and S16). However, subgroup analysis failed to significantly reduce heterogeneity and change results.

## Discussion

In this meta-analysis, by analyzing the results of ΔFVC, Δ%FVC, Δ6MWT, and Δ%DLco, NAC plus pirfenidone is unlikely to exhibit greater efficacy compared to pirfenidone monotherapy in the treatment of IPF. The initial proposal for NAC as a treatment for pulmonary fibrosis was based on findings from an animal model. NAC has been shown to enhance glutathione synthesis, regulate lysine oxidase activity, inhibit epithelial-mesenchymal transformation, and ultimately reduce the presence of oxygen free radicals and provide antioxidant effects [11, 31–33]. The efficacy of NAC monotherapy in the treatment of IPF has been controversial [34–36]. The meta-analysis conducted by Sun et al. [37] revealed that NAC had a significant impact on reducing vital capacity and Δ6MWT. However, it did not significantly reduce Δ%DLco, ΔFVC, adverse events, or mortality. Previous cohort studies and case controls have demonstrated that NAC combined with other medications has a certain efficacy in treating IPF [11–17]. In previous studies, 5 articles were cohort and case–control studies comparing the treatment of pirfenidone puls NAC versus pirfenidone monotherapy for IPF [13–17]. The conclusions reached by these articles are inconsistent. Shi H et al. [38] systematically reviewed these 5 articles and did not find that pirfenidone plus NAC had better benefits in the treatment of IPF. Nevertheless, a phase 3 RCT conducted by Sakamoto et al. [18] in Japan found that NAC plus pirfenidone did not differ from pirfenidone monotherapy in terms of Δ%DLco, Δ6MWD, and progression-free survival in IPF treatment, and ΔFVC decreased more significantly. A Phase 2 RCT in Europe by Behr J et al. [19] showed that oral NAC plus pirfenidone was unlikely to have clinical benefit. Related RCTs have also been conducted in China, but with relatively high risk of bias (Table 3). Wen JY et al. and Zhou XD et al. showed that NAC plus pirfenidone was effective in the treatment of IPF [22, 23]. However, the results of Zhao HM et al. showed that it had no better efficacy [24]. But a common shortcoming of these studies is the small number of patients. According to the PANTHER study [35], NAC may be effective in IPF treatment. However, subsequent analysis revealed its benefits primarily for patients with T/T genotypes, while patients with C/C genotypes may not experience the same benefits and could potentially experience disease progression. The study speculates that genes encoding T-cell interaction proteins(TOLLIP; Rs3750920) may influence the response to NAC [35, 36]. This may explain why some studies have shown that NAC is effective in treating IPF, but more relevant studies are needed to confirm this.

**Table 3:** RCT risk assessment using the Cochrane collaboration risk of bias tool

Reference	Random Sequence Generation^①^?	Allocation Concealment^②^?	Blinding of Participants and Personnel^③^?	Incomplete Outcome Data^④^?	Selective Reporting^⑤^?	Other Biases^⑥^?	Risk of Bias
Sakamoto (2021) [18]	+	+	-	+	+	+	High
Behr (2016) [19]	+	+	+	+	+	+	Low
Wen (2019) [22]	+	-	-	-	+	-	High
Zhou (2021) [23]	+	-	-	+	+	+	High
Zhao (2023) [24]	+	-	-	+	+	+	High

①Describe the method used to generate the allocation sequence in sufficient detail to allow an assessment of whether it should produce comparable groups

②Describe the method used to conceal the allocation sequence in sufficient detail to determine whether intervention allocations could have been foreseen in advance of, or during, enrolment

③Describe all measures used, if any, to blind study participants, personnel and outcome assessors from knowledge of which intervention a participant received

④Describe the completeness of outcome data for each main outcome, including attrition and exclusions from the analysis

⑤State how the possibility of selective outcome reporting was examined by the review authors, and what was found

⑥State any important concerns about bias not addressed in the other domains in the tool. The other bias mainly refers to the existence of at least one of the important sources of bias, such as the existence of potential sources of bias related to the specific study design used; Claims of deception; or there are some other problems

This meta-analysis showed that NAC plus pirfenidone was not more effective than pirfenidone monotherapy in the treatment of IPF in terms of at least one side effect, severe side effects, gastrointestinal effects, skin effects, and mortality rates. The side effects of NAC plus pirfenidone in the treatment of IPF have often been reported in different studies. The prevailing view is that the deaths are not related to medical treatment and are mostly the result of an exacerbation of the disease or other causes. Furthermore, severe side effects were predominantly unrelated to the administered treatment. Certain gastrointestinal effects were found to be related to the treatment, while others were not. However, these effects were generally mild to moderate in severity and could be alleviated through symptomatic treatment. The PANORAMA study indicated that the treatment group exhibited a higher incidence of photosensitivity compared to the control group, which was hypothesized to be associated with NAC [19]. Nevertheless, this meta-analysis did not reveal any significant difference in this regard [18, 19, 22–24]. These findings suggest that NAC plus pirfenidone in the treatment of IPF is relatively safe, although further confirmation is required through large-scale studies.

GRADE evidence grade evaluation results showed that moderate certainty evidence indicates that NAS plus pirfenidone as compared to pirfenidone monotherapy for IPF may not reduce the incidence of skin effects and mortality. Low certainty evidence indicates that NAS plus pirfenidone as compared to pirfenidone monotherapy for IPF may not improve Δ%DLco, ΔFVC, Δ%FVC, and Δ6MWT. Extremely low certainty indicates that NAS plus pirfenidone as compared to pirfenidone monotherapy for IPF may not improve ΔFVC, Δ%FVC, and Δ6MWT (Table 4) [25, 26].

**Table 4:** GRADE evidence quality evaluation of outcomes

Outcome	Risk of Bias^①^	Inconsistency^②^	Indirectness^③^	Imprecision^④^	Publication Bias^⑤^	Level of Evidence
ΔFVC	− 1	-2	0	0	0	Extremely low
Δ%FVC	− 1	-2	0	0	-1	Extremely low
Δ6MWT	− 1	-2	0	0	0	Extremely low
Δ%DLco	− 1	-1	0	0	0	Low
at least one side effects	− 1	0	0	0	-1	Low
severe side effects	− 1	0	0	0	-1	Low
gastrointestinal effects	− 1	0	0	0	-1	Low
skin effects	− 1	0	0	0	0	Moderate
mortality rates	− 1	0	0	0	0	Moderate

− 1 (Downgrade by 1 level); 0 (no downgrade)

①Limitations in study design or execution; Most of the information comes from medium and high risk studies

②The effect size and direction of each study were inconsistent/confidence interval overlap was small/heterogeneity test P-value was small, I^2^ > 50%

③Indirect evidence

④Insufficient sample size/The confidence interval is not narrow enough

⑤Funnel plot asymmetry/suspected large publication bias

This study also exhibits evident limitations, including small sample sizes, limited geographical coverage, low statistical power and publication bias, despite being RCTs. Moreover, there are no high certainty outcomes in this paper; variations in the administration of NAC were observed across different studies, with two studies employing inhaled NAC, leading to higher concentrations of NAC in the pulmonary system. Furthermore, the data pertaining to study outcomes is incomplete, with certain studies lacking partial PFT data and some studies lacking information on side effects. These observations underscore the necessity for high-quality, large-sample RCTs to furnish more robust clinical recommendations, but this is also very difficult.

## Conclusion

There is limited evidence that NAC plus pirfenidone is not more beneficial than pirfenidone monotherapy in the treatment of IPF in terms of ΔFVC, Δ%FVC, Δ6MWT, Δ%DLco, at least one side effect, severe side effects, gastrointestinal effects, skin effects, and mortality rates. Consequently, until more robust evidence becomes available, it is not advisable to routinely administer NAC plus pirfenidone for IPF. However, due to low statistical power and the existence of heterogeneity, objective explanations are also needed.

### Supplementary Information

**Additional file 1: Table S1.** PRISMA 2020 item checklist.

**Additional file 2: Table S2.** PRISMA 2020 for abstracts checklist.

**Additional file 3: Figure S1.** ΔFVC funnel plot.

**Additional file 4: Figure S2.** Δ%FVC funnel plot.

**Additional file 5: Figure S3.** Δ6MWT funnel plot.

**Additional file 6: Figure S4.** Δ%DLco funnel plot.

**Additional file 7: Figure S5.** Side effects funnel plot.

**Additional file 8: Figure S6.** Severe side effects funnel plot.

**Additional file 9: Figure S7.** Gastrointestinal effects funnel plot.

**Additional file 10: Figure S8.** Skin effects funnel plot.

**Additional file 11: Figure S9.** Mortality rates funnel plot.

**Additional file 12: Figure S10.** ΔFVC forest plot.

**Additional file 13: Figure S11.** Δ%DLco forest plot.

**Additional file 14: Figure S12.** Side effects forest plot.

**Additional file 15: Figure S13.** Severe side effects forest plot.

**Additional file 16: Figure S14.** Gastrointestinal effects forest plot.

**Additional file 17: Figure S15.** Skin effects forest plot.

**Additional file 18: Figure S16.** Mortality rates forest plot.

## Data Availability

The datasets used and/or analyzed during the current study are available from the corresponding author on reasonable request.
