# Supplementary material for: Efficacy of N-acetylcysteine plus pirfenidone in the treatment of idiopathic pulmonary fibrosis: a systematic review and meta-analysis
Source: BMC Pulm Med. 2023 Nov 29;23:479. doi: 10.1186/s12890-023-02778-w (PMC10685588; doi:10.1186/s12890-023-02778-w)
Supplement: Supplementary file 2 — Additional file 2: Table S2. PRISMA 2020 for abstracts checklist. [file 12890_2023_2778_MOESM2_ESM.docx]

| Section and topic | Item # | Checklist item |
| --- | --- | --- |
| **Title** |  |  |
| Title  **Background** | 1 | Identify the report as a systematic review. |
| Objectives  **Methods** | 2 | Provide an explicit statement of the main objective(s) or question(s) the review addresses. |
| Eligibility criteria | 3 | Specify the inclusion and exclusion criteria for the review. |
| Information sources | 4 | Specify the information sources (e.g. databases, registers) used to identify studies and the date when each was last searched. |
| Risk of bias | 5 | Specify the methods used to assess risk of bias in the included studies. |
| Synthesis of results  **Results** | 6 | Specify the methods used to present and synthesise results. |
| Included studies | 7 | Give the total number of included studies and participants and summarise relevant characteristics of studies. |
| Synthesis of results  **Discussion** | 8 | Present results for main outcomes, preferably indicating the number of included studies and  participants for each. If meta-analysis was done, report the summary estimate and confidence/credible interval. If comparing groups, indicate the direction of the effect (i.e. which group is favoured). |
| Limitations of evidence | 9 | Provide a brief summary of the limitations of the evidence included in the review (e.g. study risk of bias, inconsistency and imprecision). |
| Interpretation  **Other** | 10 | Provide a general interpretation of the results and important implications. |
| Funding | 11 | Specify the primary source of funding for the review. |
| Registration | 12 | Provide the register name and registration number. |

Table S2:PRISMA 2020 for abstracts checklist
