# Supplementary figures and images for: Efficacy of N-acetylcysteine plus pirfenidone in the treatment of idiopathic pulmonary fibrosis: a systematic review and meta-analysis
Source: BMC Pulm Med. 2023 Nov 29;23:479. doi: 10.1186/s12890-023-02778-w (PMC10685588; doi:10.1186/s12890-023-02778-w)

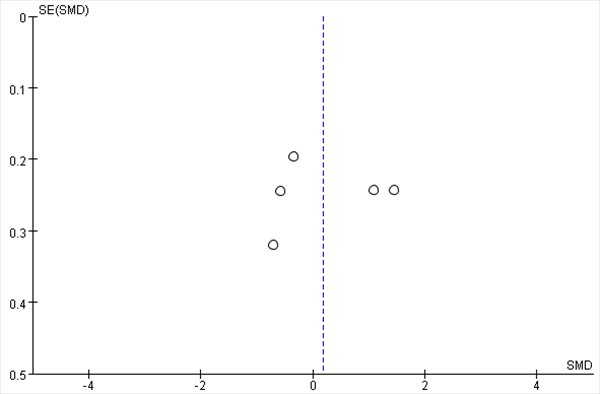

Supplement: Supplementary file 3 — Additional file 3: Figure S1. ΔFVC funnel plot. [file 12890_2023_2778_MOESM3_ESM.tif]

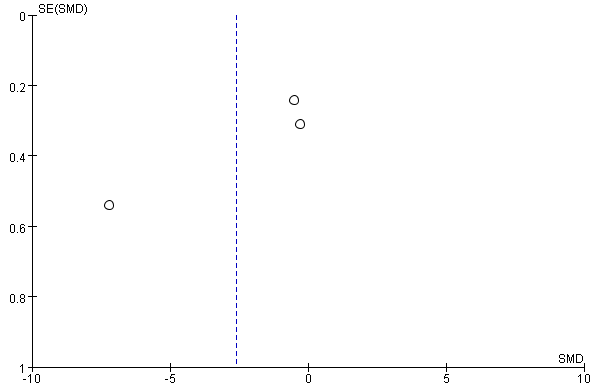

Supplement: Supplementary file 4 — Additional file 4: Figure S2. Δ%FVC funnel plot. [file 12890_2023_2778_MOESM4_ESM.tif]

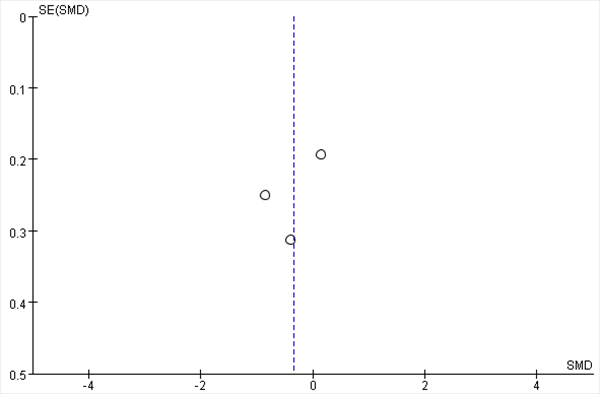

Supplement: Supplementary file 5 — Additional file 5: Figure S3. Δ6MWT funnel plot. [file 12890_2023_2778_MOESM5_ESM.tif]

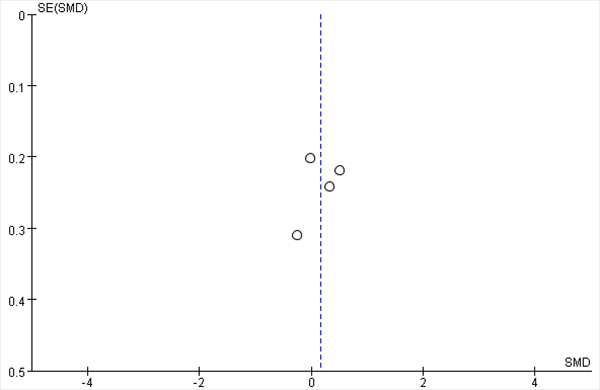

Supplement: Supplementary file 6 — Additional file 6: Figure S4. Δ%DLco funnel plot. [file 12890_2023_2778_MOESM6_ESM.tif]

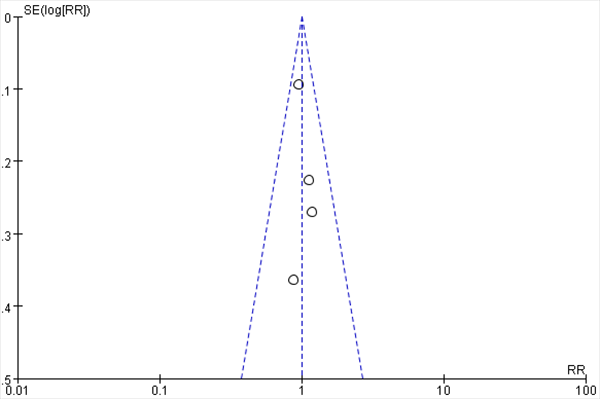

Supplement: Supplementary file 7 — Additional file 7: Figure S5. Side effects funnel plot. [file 12890_2023_2778_MOESM7_ESM.tif]

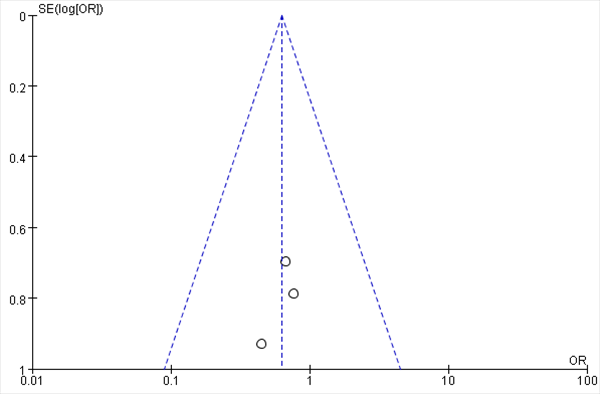

Supplement: Supplementary file 8 — Additional file 8: Figure S6. Severe side effects funnel plot. [file 12890_2023_2778_MOESM8_ESM.tif]

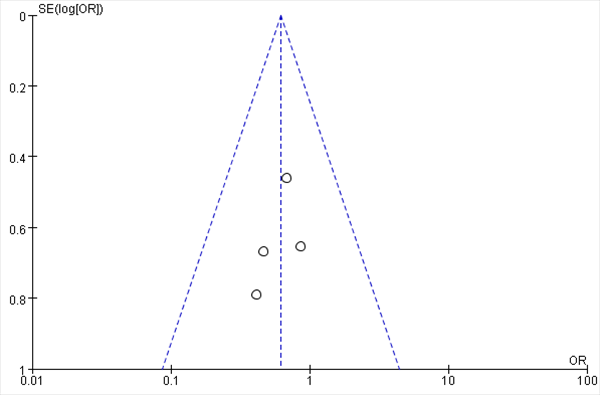

Supplement: Supplementary file 9 — Additional file 9: Figure S7. Gastrointestinal effects funnel plot. [file 12890_2023_2778_MOESM9_ESM.tif]

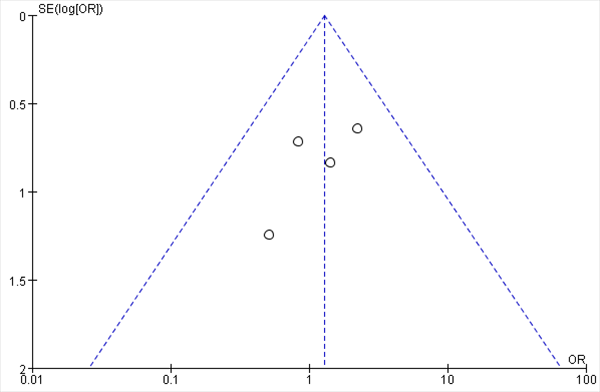

Supplement: Supplementary file 10 — Additional file 10: Figure S8. Skin effects funnel plot. [file 12890_2023_2778_MOESM10_ESM.tif]

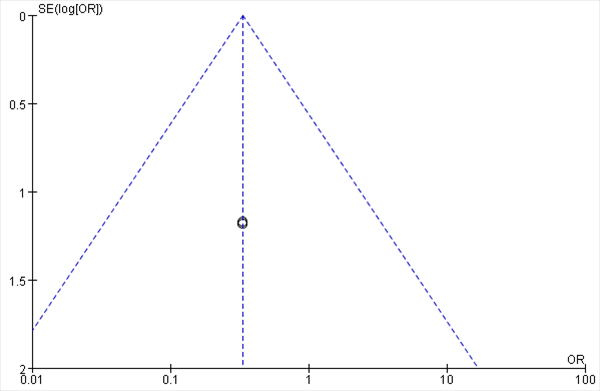

Supplement: Supplementary file 11 — Additional file 11: Figure S9. Mortality rates funnel plot. [file 12890_2023_2778_MOESM11_ESM.tif]

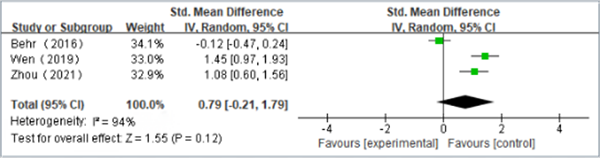

Supplement: Supplementary file 12 — Additional file 12: Figure S10. ΔFVC forest plot. [file 12890_2023_2778_MOESM12_ESM.tif]

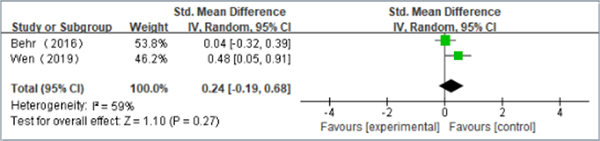

Supplement: Supplementary file 13 — Additional file 13: Figure S11. Δ%DLco forest plot. [file 12890_2023_2778_MOESM13_ESM.tif]

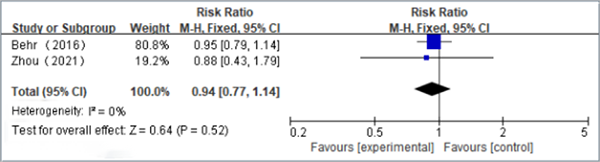

Supplement: Supplementary file 14 — Additional file 14: Figure S12. Side effects forest plot. [file 12890_2023_2778_MOESM14_ESM.tif]

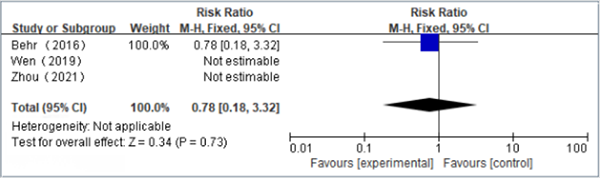

Supplement: Supplementary file 15 — Additional file 15: Figure S13. Severe side effects forest plot. [file 12890_2023_2778_MOESM15_ESM.tif]

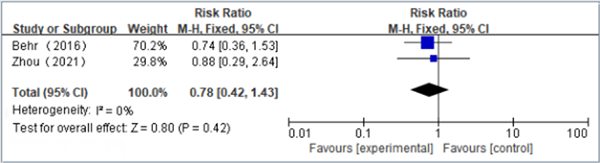

Supplement: Supplementary file 16 — Additional file 16: Figure S14. Gastrointestinal effects forest plot. [file 12890_2023_2778_MOESM16_ESM.tif]

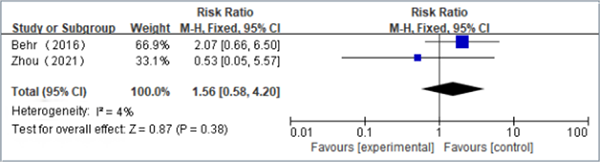

Supplement: Supplementary file 17 — Additional file 17: Figure S15. Skin effects forest plot. [file 12890_2023_2778_MOESM17_ESM.tif]

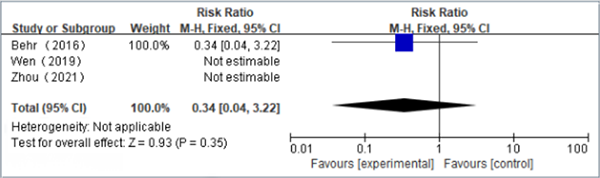

Supplement: Supplementary file 18 — Additional file 18: Figure S16. Mortality rates forest plot. [file 12890_2023_2778_MOESM18_ESM.tif]
